# Optimizing cognitive-behavioral therapy for social anxiety disorder and understanding the mechanisms of change: Study protocol for a randomized factorial trial

**DOI:** 10.1016/j.invent.2021.100480

**Published:** 2021-11-10

**Authors:** Rodrigo C.T. Lopes, Dajana Šipka, Tobias Krieger, Jan Philipp Klein, Thomas Berger

**Affiliations:** a Department of Clinical Psychology and Psychotherapy, University of Bern, Fabrikstrasse, 8, 3012 Bern, Switzerland; b Department of Psychiatry, Psychosomatics and Psychotherapy, Luebeck University, Luebeck, Germany

**Keywords:** Internet-based cognitive-behavioral therapy, Social anxiety, Factorial design, Mechanisms of change

## Abstract

**Background:**

Social anxiety disorder (SAD) is characterized by a marked fear of negative evaluation in social situations and significant impairments. Even with the most effective treatments, remission rates are around 50%. An important reason for the limited effectiveness of treatments is the lack of evidence-based explanation of how treatments work and what their active ingredients might be. An approach to unpack the active ingredients and mechanisms of treatment is the factorial design.

**Objectives:**

The study is a factorial trial aiming (1) to examine the main effects and interactions for the four main treatment components of internet-based cognitive-behavioral therapy (ICBT) for SAD (i.e., psychoeducation, cognitive restructuring, attentional training, and exposure) and (2) to examine whether and which change mechanisms mediate the relationship between treatment components and symptom reduction.

**Methods:**

A total of 464 adults diagnosed with SAD will be randomized to one of 16 conditions containing combinations of the treatment components. The primary endpoint is SAD symptomatology at eight weeks. Secondary endpoints include symptoms of depression and anxiety, quality of life, and negative effects. Hypothesized change mechanisms are the increase of knowledge about SAD, the decrease of dysfunctional cognitions, the decrease of self-focused attention, and the decrease of avoidance and safety behaviors.

**Discussion:**

A better understanding of the differential efficacy of treatment components and mechanisms of treatment underlying ICBT for SAD might inform clinicians and researchers to plan more potent and scalable treatments.

**Trial registration:**

clinicaltrials.gov (NCT04879641) on June, 11th 2021. https://clinicaltrials.gov/ct2/show/NCT04879641.

## Highlights

• Internet-based cognitive-behavioral therapy (ICBT) allows us to disentangle treatment components of complex treatment packages.
• Factorial trials are a suitable design to understand main and interaction effects of treatment components and the mechanisms of change.
• Our program for social anxiety disorder consists of four components: psychoeducation, cognitive restructuring, attention training, and exposure.
• We will test the effects for the four treatment components and the mediation of hypothesized mechanisms of change on outcomes.
• Hypothesized mechanisms of change are knowledge gain of SAD, decrease of dysfunctional social cognitions, decrease of self-focused attention, decrease of avoidance and safety behaviors

## 1 Introduction

Social anxiety disorder (SAD) is characterized by a marked and persistent fear of negative evaluation in social situations (American Psychiatric Association, 2013) and is a prevalent and disabling disorder across the globe (Stein et al., 2017). Although effective treatments such as psychotherapy and pharmacotherapy are available, far from all individuals suffering from SAD seek and eventually find help (Dalrymple and Zimmerman, 2011). Internet interventions offer many potential benefits, such as providing broader and easier access to empirically supported treatments affordably and conveniently.

The efficacy of internet interventions such as guided self-help interventions has been demonstrated for a variety of mental disorders in many randomized controlled trials (RCTs) and meta-analyses (Andersson et al., 2014; Andersson et al., 2019b; Carlbring et al., 2018; Karyotaki et al., 2017). SAD is probably the disorder for which internet-based guided self-help treatments have the most robust empirical support (Hedman et al., 2016). In this treatment format, patients work their way through a structured self-help program, typically based on CBT manuals (Clark, 2001; Clark and Wells, 1995), and therapists (also referred to as coaches or guides) assist and support them via a secure e-mail system. Overall, the vast majority of the RCTs investigating such Internet-based cognitive-behavioral treatments (ICBT) for SAD reported substantial reductions of social anxiety symptoms (Andersson et al., 2019a; Boettcher et al., 2013) and cost-effectiveness (Hedman et al., 2011). However, and as with conventional face-to-face treatments (Loerinc et al., 2015), there is still much room for improving the efficacy of ICBT for SAD as a considerable number of patients do not recover fully after treatment. The number of participants fulfilling the criterion of clinically significant change (Jacobson and Truax, 1991) at the end of ICBT ranges between 36 and 56% across studies on ICBT for SAD (Boettcher et al., 2013).

An important reason for the limited efficacy of face-to-face and ICBT for SAD is the limited understanding of how these treatments work and what their active ingredients might be. Various reviews convincingly argued that the active ingredients of CBT need to be identified so that therapy can be made more efficacious and probably also briefer (e.g., Holmes et al., 2014; Kazdin, 2017). Identifying the active ingredients requires the use of rigorous study designs that test the presence or absence of individual therapeutic elements rather than conventional parallel-group randomized controlled trials (RCTs). Conventional RCTs are the gold standard for determining if the intervention package works by establishing the relative efficacy of one treatment intervention versus a control group (e.g., another treatment package, attention control, wait-list). However, RCTs have limitations in identifying active ingredients because they usually only compare the overall effect of an entire intervention package. Current evidence-based psychological treatments for SAD, such as CBT, are made of multiple components, including psychoeducation, cognitive restructuring, attention training, and exposure. Each of these treatment components can (a) contribute to a greater or lesser extent to the effect of the treatment package, (b) act via distinct mechanisms, and (c) act via synergistic or antagonistic interactions (Collins, 2018).

As for the specific case of SAD, little effort has been made to answer the question of the differential effects of treatment components in face-to-face settings. Evidence for differential effects of specific treatment components usually comes from underpowered clinical trials with little control over treatment integrity, and results are inconsistent (e.g., Hope et al., 1995; Mattick et al., 1989; Nortje and Posthumus, 2012). The most reliable evidence so far comes from meta-analyses, but the conclusions are still not consistent. For instance, Powers et al. (2008) found that cognitive and behavioral interventions for SAD combined were not significantly more effective than cognitive treatments alone or exposure treatments alone. Also, no significant differences were found in direct comparisons of cognitive techniques alone and exposure alone (Feske and Chambless, 1995; Gil et al., 2001; Powers et al., 2008). These findings conflict with other meta-analytic evidence showing that exposure-based interventions alone yielded the largest effect size, whether alone or combined with cognitive restructuring (Gould et al., 1997). Besides the discrepant findings, research has failed to look into the active components of treatments separately (Acarturk et al., 2009). Thus, it is unclear which intervention components work and which do not and which ones work particularly well together. In line with this, how or why well-studied interventions for SAD produce change is mostly unknown.

The factorial experiment is an efficient and economical way of studying the individual and combined effects of sets of intervention components (Collins et al., 2014; Watkins and Newbold, 2020). However, factorial designs are still rare in psychotherapy research. One of the reasons is that in traditional psychotherapy, it is challenging to clearly demarcate treatment components and avoid an unwanted drift from the treatment protocol by therapists. Standardization and the avoidance of spillover effects (e.g., avoiding therapists using techniques from other treatment components) are essential for a successful factorial experiment. With the advent of internet-delivered treatments, the possibility to successfully realize factorial designs has improved. In this new treatment format, the intervention content can be standardized, and treatment integrity (the degree to which an intervention is implemented as intended) can be controlled (Collins, 2018; Watkins et al., 2016).

Although adding a factor to a factorial experiment does not require the same relatively large increase of the number of participants as an additional treatment arm would require in an RCT (see below; Collins et al., 2014), factorial trials still need quite large sample sizes to have sufficient power. With internet interventions, it is much easier to conduct large trials than in conventional psychotherapy research, with some clinical trials with more than 1000 participants (e.g., Klein et al., 2016), which is one reason why this field has developed at a fast pace (Andersson, 2015). Due to the possibility to control the delivery of standardized treatment components and to run trials with large sample sizes, several research groups (including our own) have recently started to conduct factorial trials to identify the active ingredients of internet interventions (e.g., Berg et al., 2020b; Bur et al., 2021; Watkins et al., 2016). Factorial trials have also been recommended to understand the mechanisms of change because they “provide direct evidence about the effects and interactions of individual components within a treatment package” (Watkins and Newbold, 2020, p. 429).

### 1.1 Objectives

The primary objective of this trial is to investigate the active ingredients of ICBT for SAD by testing the main effects and interactions for the four main treatment components (i.e., psychoeducation, cognitive restructuring, attention training, and exposure) on primary (i.e., decrease in social anxiety symptoms) and on secondary outcomes (i.e., decrease in depressive symptoms, decrease in general anxiety, increase of quality of life, and client satisfaction). Furthermore, we also aim to investigate the effects of each treatment component on hypothesized change mechanisms and explore whether and which change mechanisms mediate the effect of the treatment components on symptom reduction. The specific secondary objectives (1) to investigate whether the specific mechanisms (i.e., knowledge gain of SAD, decrease of dysfunctional social cognitions, decrease of self-focused attention, decrease of avoidance and safety behaviors) mediate the effect of the treatment components on primary and secondary outcomes, and (2) to address additional exploratory research questions, including examining the negative effects of the treatment components and potential moderators of treatment outcome.

## 2 Methods

### 2.1 Study design

The study is a single-center, block randomized, balanced factorial trial with four treatment components (experimental factors), each evaluated at two levels (presence vs absence), resulting in 16 conditions (2 × 2 × 2 × 2; see Table 1). Although there are 16 experimental conditions, this study should not be considered a 16-arm RCT (Collins, 2018). The purpose of the factorial experiment is not to compare the 16 conditions to each other but to estimate the main effects of the four treatment components and interactions between the components. Each estimation of the main effects and interactions is based on all the conditions and, therefore, on all participants. For example, the main effect of the cognitive restructuring component will be estimated by comparing the mean of the experimental conditions in which the cognitive restructuring component is present (5, 6, 7, 8, 13, 14, 15, 16 in Table 1) vs the mean of the experimental conditions, in which cognitive restructuring is absent (1, 2, 3, 4, 9, 10, 11, 12 in Table 1). To calculate a two-way interaction (i.e. effect of one component depending on the level of the other factor), one has to calculate the difference between the average effect of one component at the two levels of the other component (present vs absent) and then averaging over all other factors. For a detailed explanation, see Collins (2018).

**Table 1:** Experimental conditions of the factorial design, with the presence (yes) and absence (no) of each component.

Condition	Psychoeducation	Cognitive restructuring	Attention training	Exposure
1 WL	No	No	No	No
2	No	No	No	Yes
3	No	No	Yes	No
4	No	No	Yes	Yes
5	No	Yes	No	No
6	No	Yes	No	Yes
7	No	Yes	Yes	No
8	No	Yes	Yes	Yes
9	Yes	No	No	No
10	Yes	No	No	Yes
11	Yes	No	Yes	No
12	Yes	No	Yes	Yes
13	Yes	Yes	No	No
14	Yes	Yes	No	Yes
15	Yes	Yes	Yes	No
16 full	Yes	Yes	Yes	Yes

WL = Wait-list condition. For ethical reasons, participants randomized to condition 1 will be offered treatment after eight weeks.

### 2.2 Participants

A total of 464 participants with a SAD diagnosis will be included in the study, with 29 participants each assigned to one of the 16 conditions. Participants who return the informed consent will be included in the study if they (1) are 18 years or older; (2) have access to the internet and to a smartphone, PC or tablet; (3) have sufficient knowledge of German; (4) exceed predefined cut-off scores out of two social anxiety measures (22 points on the Social Phobia Scale or 33 points on the Social Interaction Anxiety Scale; SPS & SIAS; German version: Stangier et al., 1999); (5) fulfill the diagnostic criteria of SAD according to a diagnostic telephone interview; (6) in the case of taking psychiatric medication, the treatment is stabilized over one month.

Candidates will be excluded from the study if they (1) score two or higher on the suicide item of the PHQ-9 or show active suicidal plans in the diagnostic telephone interview; (2) have other highly impairing comorbid psychiatric conditions (i.e., history of psychotic or bipolar disorder) and (3) undergo another psychological treatment at the beginning of the study.

### 2.3 Recruitment

Participants will be recruited using reports in newspapers, flyers, through internet forums, social media (e.g., Facebook), via our study website (https://selfhelp1.psy.unibe.ch/shyne/homepage_interessierte) and our research hub website for internet interventions (http://www.online-therapy.ch/) in German-speaking countries. The link to the study website will also be publicized using Facebook Ads and the Google Ads tool.

### 2.4 Treatment

The internet-based self-help program (Shyne) is based on the well-established cognitive-behavioral treatment for social anxiety disorder by Clark and Wells (1995). It has been proven efficacious in previous studies in our research hub (Berger et al., 2009, Berger et al., 2011; Schulz et al., 2016; Stolz et al., 2018) as well as in previous studies from other universities and countries (e.g., Boettcher et al., 2012; Chen et al., 2020; Kählke et al., 2019; Kishimoto et al., 2016; Lin et al., 2020). The Shyne program consists of the following four treatment components:

1) *Psychoeducation*: This treatment module delivers (1) detailed evidence-based information on SAD with a focus on maintaining processes (e.g., the vicious cycle of negative thoughts and emotions, cognitions, and behaviors associated with the maintenance of SAD) and (2) a brief overview of the evidence-based CBT strategies to overcome SAD (i.e., psychoeducation about the principles behind cognitive restructuring, attention training and exposure). Additionally, participants are asked to write about their anxiety-inducing situations as well as thoughts, feelings and possible avoidance behaviors associated with the described situations. Participants are encouraged to develop an individual model of their social anxiety symptoms based on the information provided.
2) *Cognitive restructuring*: In this treatment module, participants are instructed to identify and modify dysfunctional and negatively biased assumptions. It includes a thought diary to track negative beliefs in daily routine and exercises to formulate helpful and adaptive thoughts.
3) *Attention training*: In this treatment module, participants are trained to reduce self-focused and biased attention. Audio, video and text-based exercises in which participants learn to intentionally direct the attention away from themselves (i.e., less private self-consciousness) and to be less alert to potentially dangerous external social stimuli (i.e., less public self-consciousness; Duval and Wicklund, 1972; Fenigstein et al., 1975).
4) *Exposure*: In this treatment module, participants are instructed to plan and track in vivo exposures using an exposure diary. Participants are also advised to reduce safety behaviors, overt or covert acts such as avoiding eye contact or rehearsing sentences to prevent a feared outcome.

The four treatment components have the same content throughout all conditions but are slightly changed to make sense when combined in a particular treatment condition. Short, specific psychoeducation is also given as an introduction to each component (e.g., an explanation about the relationship between cognition and emotions in the cognitive restructuring component). Moreover, all participants, independent of the condition, get an introduction module at the beginning and a conclusion module at the end. The introduction module gives an overview of the program and informs the participants about how they can work with it. As a motivational strategy, participants are asked to list their personal goals with the treatment and the expected life changes after overcoming the symptoms of social anxiety. The introduction module has the same content for all conditions. In the conclusion modules, participants are provided with a summary of the steps they should follow and repeat after the program termination. They are also asked to summarize the exercises, thoughts, and behaviors that helped them cope with relapses and were generally perceived as the most helpful. We wrote sixteen different conclusion modules since the summary and recommended repetition are different for every condition.

Shyne can be accessed through a secure website from various devices such as PCs, tablets and smartphones, with each participant having a password-protected account. The program will automatically record participants' usage of Shyne, allowing for an automated measure of treatment adherence and treatment dosage.

### 2.5 Minimal guidance

The role of the guides is to reinforce independent program use and keep up the participants' motivation and adherence. Participants will also have the opportunity to send questions to their assigned guide throughout the program if they have difficulties with the program. As in other studies (Berger et al., 2009, Berger et al., 2011; Schulz et al., 2016; Stolz et al., 2018), the guides monitor the progress of the participants in the program and contact them via a secured text-based messaging system once a week to provide feedback and encourage further engagement. In case of non-adherence, the guides will remind the participants of the importance of reading the material and doing the exercises proposed by Shyne. Guides will also encourage repetition, especially in conditions with fewer components, to ensure dosage equivalence across all conditions. Minimal guidance will be provided by advanced master's students in clinical psychology and psychotherapy. Guides will be randomly assigned to participants across the conditions.

Training and weekly supervision of the master students for the diagnostic interview and guidance will be provided by the first, second and last author (two licensed psychotherapists and experienced in internet-based guided self-help treatments, and a PhD student in clinical psychology). In the supervision, the chats between participants and guides are reviewed, and it is made sure that guidance is being kept at a minimal level.

### 2.6 Procedures

The study procedures have been approved by the Ethics Committee of the Canton Bern (KEK Bern 2020-02952) and were registered on clinicaltrials.gov (NCT04879641). After receiving the study information and signing the informed consent, the candidates will be screened for eligibility with self-report measures. Potential participants who fulfill the inclusion criteria will be interviewed by telephone to ascertain whether they meet the criteria for SAD. After checking the inclusion criteria, the eligible participants will be randomized with equal probability to one of the 16 treatment conditions. A permuted block randomization schedule will be created using the *blockrand* package in R (Snow, 2020). The random allocation will be concealed to the investigators and done by the in-built randomization module in the REDCap software (Harris et al., 2009, Harris et al., 2019). Assessments of the primary and secondary outcomes and hypothesized mediators are taken at (1) baseline (pre-treatment), (2) at four weeks after randomization (mid-treatment), (3) eight weeks after randomization (post-treatment), and (4) at six months after randomization (follow-up).

**Fig. 1:**
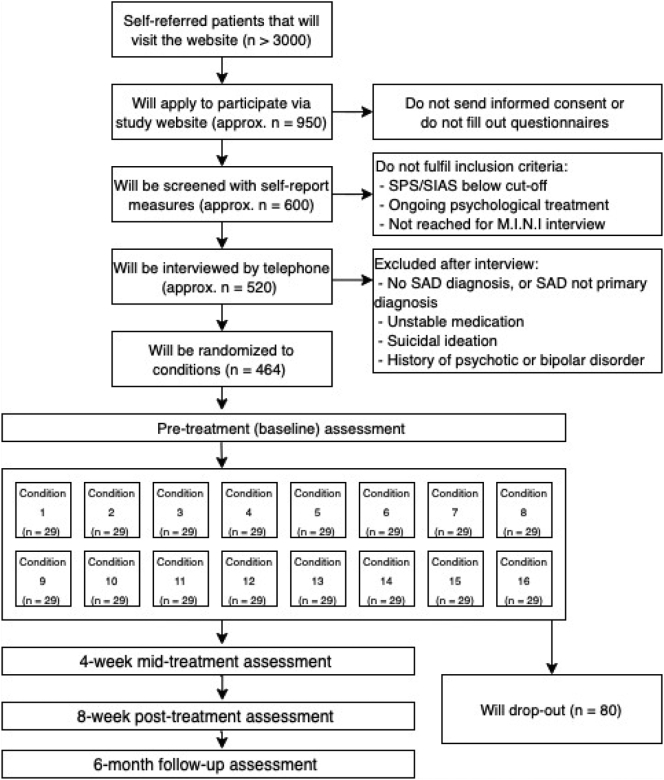
Design of the study and expected participants' flow.

As shown in Table 1, all conditions contain at least one component of ICBT for SAD, except for condition number 1, which is a wait-list control group. For ethical reasons, participants randomly assigned to the wait-list control group will receive the full treatment (i.e., condition 16) after the post-assessment. Participants randomized to one of the active treatment conditions can use the full treatment after the follow-up assessment (Fig. 1).

### 2.7 Instruments

Table 2 summarizes the instruments and time points of assessment that will be used.

**Table 2:** Variables, instruments and time points of assessment.

Dimension	Instrument	Abbreviation	Authors (German version)	Timepoints
*Primary outcome measure*				
Social anxiety symptoms	Social Phobia Scale & Social Interaction Anxiety Scale	SPS & SIAS	Stangier et al. (1999)	Pre, Mid, Post, FU

*Secondary outcome measures*				
Depressive symptoms	Patient Health Questionnaire	PHQ-9	Gräfe et al. (2004)	Pre, Mid, Post, FU
General anxiety symptoms	Generalized Anxiety Disorder Scale	GAD-7	Löwe et al. (2008)	Pre, Mid, Post, FU
Quality of life	SF-12 Health Survey	SF-12	Gandek et al. (1998)	Pre, Mid, Post, FU
Client satisfaction	Client Satisfaction Questionnaire	CSQ-8	Schmidt and Wittmann (2002)	Post
Negative effects	Negative Effects of the Treatment & Symptom Deterioration	INEP	Ladwig et al. (2014)	Mid, Post, FU
Diagnoses	MINI. Neuropsychiatric Interview	MINI 6.0.0	Sheehan et al. (1998)	Pre, Post, FU

*Hypothesized change mechanisms*				
Knowledge of SAD	Knowledge of SAD test	KSAD	Andersson et al. (2012)	Pre, Mid, Post, FU
Dysfunctional social cognitions	Social Cognitions Questionnaire	SCQ	Stangier et al. (1997)	Pre, Mid, Post, FU
Self-focused attention	Self-Consciousness Scale	SCS	Filipp and Freudenberg (1989)	Pre, Mid, Post, FU
Fear and avoidance	Liebowitz Social Anxiety scale	LSAS-SR	Stangier and Heidenreich (2004)	Pre, Mid, Post, FU
Safety behaviors	Social Behaviors Questionnaire	SBQ	Stangier et al. (1996)	Pre, Mid, Post, FU

Notes. Pre = baseline; Mid = mid-treatment (4 weeks after baseline); Post = post-treatment (8 weeks after baseline); FU = follow-up (6 months after baseline).

#### 2.7.1 Primary outcome

SAD symptoms after eight weeks are the primary outcome of the study and will be assessed with the composite score of the Social Phobia Scale and the Social Interaction Anxiety Scale (SPS & SIAS; Mattick and Clarke, 1998; German version: Stangier et al., 1999). These two self-report questionnaires complement one another and are usually administered together. The SIAS assesses fears of social interaction (e.g., “I tense up if I meet an acquaintance in the street”), while the SPS focuses on fears of being judged by others (e.g., “I become anxious if I have to write in front of others.”). Both scales together consist of 40 items to be rated on a 5-point Likert scale (0 = “not at all” to 4 = “extremely”, ranging from 0 to 160 points, where high scores mean more general fear of social interaction). These two companion measures have been found to be valid, reliable and useful for clinical and research purposes. The German version of SIAS has an internal consistency of Cronbach's α = 0.94 (Stangier et al., 1999). The German version of the SPS also has an internal consistency of α = 0.94 (Stangier et al., 1999). They are both highly correlated with the other social anxiety measures, such as the Social Phobia and Anxiety Inventory and the Liebowitz Social Anxiety Scale (Hoyer and Margraf, 2003; Stangier et al., 1999). Stangier et al. (1999) calculated a cut-off value of 22 (for SPS) and 33 (for SIAS) as a discrimination criterion between German-speaking patients with social anxiety and different comparison groups. The composite score will be the simple average of the *z*-scores of SIAS and SPS, as recommended by Song et al. (2013) for continuous variables.

#### 2.7.2 Secondary outcomes

##### 2.7.2.1 M.I.N.I. International Neuropsychiatric Interview for DSM-IV 6.0.0 (M.I.N.I.; Sheehan et al., 1998; German version: Ackenheil et al., 1999)

M.I.N.I. is a brief structured diagnostic interview for assessing psychiatric diagnosis based on the DSM-IV. The specificity of the M.I.N.I was reported as suitable for all diagnoses (ranging from 0.72 to 0.97; 0.81 for SAD; Sheehan et al., 1997). In our study, the M.I.N.I. interview will be administered via telephone. It will assess depressive episodes, suicidality, manic and hypomanic episodes, panic disorder, agoraphobia, SAD, obsessive-compulsive disorder, post-traumatic stress disorder, substance abuse and addiction, psychosis, anorexia and bulimia nervosa and generalized anxiety disorder. The diagnosis of SAD will serve as an eligibility criterion and a secondary outcome measure (an absence of diagnosis at post-treatment and follow-up suggesting treatment success).

##### 2.7.2.2 Patient Health Questionnaire (PHQ-9; Spitzer et al., 1999; German version: Gräfe et al., 2004)

Symptoms of depression will be measured with the PHQ-9. This widely used self-report measure consists of nine questions assessing characteristic symptoms of major depression described in DSM-V distributed in nine items on a 4-point Likert scale. Higher scores indicate more severe depression. The German version of the PHQ-9 has also shown good internal consistency (Cronbach's α  = 0.88; Gräfe et al., 2004; Kroenke et al., 2010; Löwe et al., 2004).

##### 2.7.2.3 Generalized Anxiety Disorder Scale (GAD-7; Spitzer et al., 2006; German version: Löwe et al., 2008)

The GAD-7 measures seven general anxiety symptoms (i.e., feeling nervous, worrying, having trouble relaxing, restlessness, feeling annoyed or irritable, and feeling afraid that something awful might happen). Higher scores indicate more severe general anxiety symptoms. The internal consistency of the GAD-7 is good in both the original and German versions (Löwe et al., 2008; Spitzer et al., 2006).

##### 2.7.2.4 Short-Form Health Survey-12 (SF-12; Ware et al., 1996; German version: Gandek et al., 1998)

Quality of life is assessed with the SF-12. Its two subscales measure the physical and mental aspects of health-related quality of life. The SF-12 shows good psychometric properties (e.g., internal consistency of α = 0.83) and is equivalent to the long-form, the SF-36 (Gandek et al., 1998; Ware et al., 1996).

##### 2.7.2.5 Client Satisfaction Questionnaire (CSQ-8; Attkisson and Zwick, 1982; German version: Schmidt and Wittmann, 2002)

The CSQ-8 is a self-report questionnaire that assesses the general level of satisfaction with the service received. It was developed to measure satisfaction with inpatient treatment. The original version shows good internal consistency (Cronbach's α = 0.91; Attkisson and Zwick, 1982). In this study, we will use a version that was adapted for internet-based treatments.

##### 2.7.2.6 Negative Effects of the Treatment (INEP; Ladwig et al., 2014)

The INEP assesses any adverse effects on social, intrapersonal or work-related situations and whether they are attributed to the intervention. As in other studies, the INEP will be slightly adapted for use within internet-based interventions. The original scale was developed and validated in German and showed good internal consistency (Cronbach's α = 0.86; Ladwig et al., 2014).

##### 2.7.2.7 Adherence

Following the suggestion of Donkin et al. (2011), a composite score to measure adherence and dosage will be created by averaging the *z*-scores of several variables: time spent in the intervention, the number of modules completed, the number of exercises completed, and the number of clicks in the intervention.

In addition, socio-demographic variables such as age, gender, country of origin, parent's country of origin, mother tongue, relationship status, educational level and employment status are assessed.

#### 2.7.3 Assessment of hypothesized mechanisms of change

**Fig. 2:**
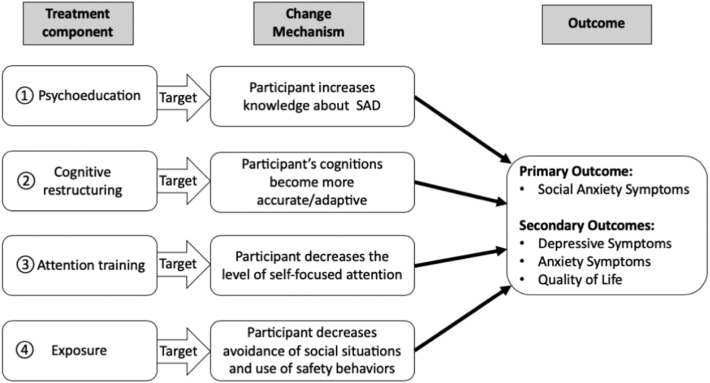
Simplified conceptual model of the effects of the four treatment components on the hypothesized change mechanisms and primary and secondary outcomes.

The secondary aim of this study is to better understand which mechanisms of change mediate the relationship between treatment components and symptom reduction. For this, we will also assess variables hypothesized to mediate change for every treatment component. Fig. 2 shows a conceptual model of the expected effects of the four treatment components on the hypothesized change mechanisms and primary and secondary outcomes.

##### 2.7.3.1 Knowledge of SAD test (KSAD; Andersson et al., 2012; Berg et al., 2020a)

The KSAD assesses basic knowledge around the condition of SAD and its treatment. It includes 11 questions, each with one correct answer (out of three possible choices). In addition, each response is rated in terms of how confident the participant is about the response with three response options (Guessing, Pretty Certain, Confident). A higher score indicates more knowledge. Questions cover the content of the psychoeducation component of the Shyne program (e.g., the definition of SAD, general principles of CBT, safety behaviors, avoidance, negative automatic thoughts, attentional shift, exposure). Scores of knowledge of SAD are calculated in two ways: (a) a total score based on the total number of correct answers and (b) a weighted total score in which certainty of answers was factored in. Reliability analyses showed a low Cronbach's alpha of α  =  0.40 for the raw scores, a high Cronbach's alpha of α  =  0.86 for the certainty ratings, and an alpha of α  =  0.56 for the weighted scores (Andersson et al., 2012).

##### 2.7.3.2 Social cognitions questionnaire (SCQ; Wells et al., 1993; German version: Stangier et al., 1996b)

The SCQ is a self-rating scale that assesses typical negative social cognitions of socially anxious individuals. It is composed of 22 items, ranging from 22 to 110, grouped in three subscales (“negative self”, “performance anxiety”, and “fear of showing bodily symptoms”). Higher scores mean more negative social cognitions. The Cronbach's α for the whole German version scale is α = 0.89.

##### 2.7.3.3 Self-Consciousness Scale (SCS; Fenigstein et al., 1975; German version: Filipp and Freudenberg, 1989)

The SCS measures self-focused attention (or self-consciousness) in two dimensions: private self-consciousness and public self-consciousness. The German version consists of 27 items, which are rated from 1 (“very rarely”) to 5 (“very often”). Higher scores indicate more self-focused attention. Both subscales have shown good internal consistency (Cronbach's α = 0.87 and α = 0.86, respectively; Hinz et al., 2010).

##### 2.7.3.4 Liebowitz Social Anxiety Scale, self-report (LSAS-SR; Baker et al., 2002; German version: Stangier and Heidenreich, 2004)

The LSAS-SR measures SAD symptoms. It comprises 24 items, divided into two subscales (anxiety and avoidance, 12 items each) scored on a Likert-type scale of four points and is rated in terms of frequency (never, occasionally, often and usually). In this study, and as a measure of a hypothesized mechanism of the change, only the avoidance subscale will be used. LSAS-SR shows good internal consistency (α = 0.96 for the total scale and α = 0.92 for the avoidance scale).

##### 2.7.3.5 Social Behaviors Questionnaire (SBQ; Clark et al., 1995; German version: Stangier et al., 1996a)

The SBQ assesses the use of safety behaviors in social situations with 27 items. The frequency of each behavior is rated on a 4-point scale (from 0 = never to 3 = always). “Avoid eye contact”, “try to control shaking”, “rehearse sentences in your mind” are examples of safety behavior assessed by the SBQ. The items on the SBQ are a mixture of discrete behaviors (e.g., hide your face, grip glasses tightly) and broad strategies (e.g., make an effort to come across well, try not to attract attention). Studies with adult populations revealed acceptable internal consistencies (Cronbach's α = 0.69).

### 2.8 Sample size

The current study is powered for the first and primary research question, i.e., the main and interaction effects of the treatment components on the decrease of social anxiety symptoms. In the a priori power analysis, we assumed that the smallest clinically relevant difference would be a small effect size of Cohen's *d* = 0.2 for the main effect of an individual treatment component or interaction between components on pre-to-post change on social anxiety symptoms. Smaller effect sizes would be of little clinical interest and value. At an α level of 0.05, a statistical power (1-Beta) of 0.80, and a correlation between measurements with around 4- and 8-weeks interval of *r* = 0.50, based on our experience with clinical trials for SAD, we need a total of 384 participants (G*Power; Faul et al., 2007). Based on the finding that in 15 out of 17 studies, dropout rates (i.e., not providing assessment data at post-treatment) for ICBT for SAD were below 13% (Boettcher et al., 2013), we conservatively estimate a dropout rate of 20% for our study (*n* = 77 participants). Thus, we aim for a sample size of 464 participants, which results in 29 individuals per treatment condition.

As mentioned above, the logic behind how an experiment is powered differs between RCTs and factorial experiments. An RCT would compare the 16 conditions to each other, and power would be reflected in the per-condition sample size. If an RCT has a small per-condition sample size, such as 29 individuals per treatment condition, it does not have enough power to detect small effect sizes. By contrast, in factorial experiments, all participants receiving a specific component (e.g., cognitive restructuring, which is present in half of the conditions, i.e., in eight conditions) can be compared to participants who do not receive that component (also eight conditions, with 29 participants per condition). That yields a sample size of *n* = 232 per component (Collins, 2018). Since our study contains only components with two levels (absent vs present), the sample size to maintain the power to detect main effects and interactions is the same (Collins, 2018). For a detailed explanation of how factorial experiments maintain power to estimate main effects and interactions, see Collins (2018).

### 2.9 Statistical analyses

Reporting will follow CONSORT E-Health standards (Eysenbach and Consort-Ehealth Group, 2011). The primary outcome is the change in the composite score of SPS & SIAS from baseline to eight weeks (post-treatment). The analyses are carried out on the basis of the intention-to-treat approach (ITT; i.e., using all randomized participants). Our primary interest is in testing the main effects and interactions. For that, we will use linear mixed models repeated measures analysis of variance (ANOVA). This approach uses all available data on each subject and does not require the imputation of missing values but estimates parameters about missing values. Furthermore, mixed models account for the correlation between the repeated measurements. Main effects and interactions are calculated based on aggregates across experimental conditions. The levels of the factors will be represented numerically by −1 (absence of a component in a condition) and +1 (presence of a component in a condition), as recommended by Collins (2018). Significance testing of dichotomous data such as diagnostic status will be conducted with chi-square tests. Sensitivity analysis will be conducted to analyze the impact of dropouts on our results.

We will test mediation of the hypothesized change mechanisms (i.e., knowledge gain of SAD, decrease of dysfunctional social cognitions, decrease of self-focused attention, decrease of avoidance and safety behaviors) of the effect of the treatment components on primary and secondary outcomes (see Fig. 2 above). We will test mediation of the hypothesized change mechanisms by using an approach that allows multiple mediators in one model, as set out by Kraemer et al. (2002).

In addition, we will explore potential moderation of the treatment components by various measured variables (i.e., age, gender, country of origin, nationality, country of parents, mother tongue, relationship status, educational level, employment status, presence of comorbid disorder, use of medication, the severity of SAD). For the analysis of potential moderators, factorial ANOVA and multiple regression analysis will be used.

## 3 Discussion

Far from all individuals suffering from SAD seek and eventually find help, and far from all SAD patients respond fully to current evidence-based treatments. Low-threshold and cost-effective internet-based interventions can easily be distributed and flexibly used, representing a promising alternative to face-to-face therapy. With an optimized internet-based intervention, a broader population of people suffering from SAD can be reached at even lower costs and more effectiveness. The results of this trial are expected to improve current evidence-based treatments for SAD and increase the number of SAD patients fully responding to ICBT. If we know more about the active ingredients of CBT for SAD, we can probably identify better and briefer strategies that trigger change processes. Thus, understanding active ingredients and change mechanisms can optimize change and “build more potent, scalable, and efficient treatments” (Watkins et al., 2016, p. 2) of SAD.

We understand the use of the factorial trial as an appropriate approach to understand the differential effects of each component of SAD. Although other sophisticated approaches exist, for instance, component individual patient data meta-analysis (e.g., Furukawa et al., 2021), the component meta-analyses are based on the indirect comparisons between different trials. Thus, there is a higher likelihood that the observed differences can be attributed not to the various components but to the differences in the settings. Furthermore, the OPTIMIZE trial is planned to have a reasonably high sample size and sufficient power to detect even small changes.

Some potential limitations of this study should be addressed. The treatment dosage may vary across conditions and be lower in those conditions with fewer components. To prevent high variations in treatment dosage, the participants are encouraged by the program and by the guides to repeat the exercise. To address this potential limitation, we will control the overall treatment dosage of each participant by assessing adherence to the program. Also, there might be a spillover effect from the psychoeducation component once we briefly explain broad change principles used in established CBT treatment (i.e., cognitive restructuring, attention training and exposure). However, we do not offer any practical indication of implementing those techniques, and we do not provide any access to the exercises introduced in the relevant components.

Finally, the measure of knowledge gain (KSAD; Andersson et al., 2012; Berg et al., 2020a) might represent a limitation since the original authors have not found satisfactory reliability. We will replicate the reliability and test-retest analysis with our sample using all time points available to re-evaluate the ability of this scale to capture change in knowledge gain.

We aim to perform mediation analyses to test the hypothesized mechanisms of change. The mere statistical mediation is not enough to ascertain a mechanism of change (Kazdin, 2007). The field needs to show a solid theoretical foundation for a specific mediator and also strong statistical association, temporality (i.e. timeline shows that intervention leads to change in mediator which leads to change in outcome, not the other way around), specificity (i.e., to prove that one particular mediator is responsible for change), gradient (i.e., dose-response relationship between mediator and outcome), consistency (across studies with different samples) and coherence with other evidence, for instance, results coming from experimental studies (Kazdin, 2007). In our study, we will be able to assess temporality, specificity and the gradient of theoretically founded mechanisms of change. Although other studies will be needed to evaluate consistency and coherence with other experimental evidence, our results might move forward theoretical debates regarding the mechanisms involved in the maintenance of SAD and what works in treatments. We estimate that this trial's results will inform the treatment of social anxiety via internet interventions and inform face-to-face treatments. At a societal level, optimizing treatment and expanding the knowledge about mechanisms of change is essential because SAD is very common and one of the costliest psychiatric conditions (e.g., Fehm et al., 2005). By determining the importance of each component to the overall efficacy of CBT treatment for SAD, we will be able to inform mental health policy decisions that would probably decrease its costs and increase its effectiveness.

## Declaration of competing interest

All the authors have no financial or scientific competing interests to declare.

## References

[bb0005] Acarturk C., Cuijpers P., van Straten A., de Graaf R. (2009). Psychological treatment of social anxiety disorder: a meta-analysis. Psychol. Med..

[bb0010] Ackenheil M., Stotz-Ingenlath G., Dietz-Bauer R., Vossen A. (1999).

[bb0015] American Psychiatric Association (2013).

[bb0020] Andersson G. (2015).

[bb0025] Andersson G., Carlbring P., Furmark T., S. O. F. I. E. Research Group (2012). Therapist experience and knowledge acquisition in internet-delivered CBT for social anxiety disorder: a randomized controlled trial. PLoS ONE.

[bb0030] Andersson G., Carlbring P., Rozental A. (2019). Response and remission rates in internet-based cognitive behavior therapy: an individual patient data meta-analysis. Front. Psych..

[bb0035] Andersson G., Carlbring P., Titov N., Lindefors N. (2019). Internet interventions for adults with anxiety and mood disorders: a narrative umbrella review of recent meta-analyses. Can. J. Psychiatry.

[bb0040] Andersson G., Cuijpers P., Carlbring P., Riper H., Hedman E. (2014). Guided internet-based vs. Face-to-Face cognitive behavior therapy for psychiatric and somatic disorders: a systematic review and meta-analysis. World Psychiatry.

[bb0045] Attkisson C.C., Zwick R. (1982). The client satisfaction questionnaire: psychometric properties and correlations with service utilization and psychotherapy outcome. Eval. Program Plann..

[bb0050] Baker S.L., Heinrichs N., Kim H.-J., Hofmann S.G. (2002). The liebowitz social anxiety scale as a self-report instrument: a preliminary psychometric analysis. Behav. Res. Ther..

[bb0055] Berg M., Andersson G., Rozental A. (2020). Knowledge about treatment, anxiety, and depression in association with internet-based cognitive behavioral therapy for adolescents: development and initial evaluation of a new test. SAGE Open.

[bb0060] Berg M., Rozental A., de Brun Mangs J., Näsman M., Strömberg K., Viberg L., Wallner E., Åhman H., Silfvernagel K., Zetterqvist M., Topooco N., Capusan A., Andersson G. (2020). The role of learning support and chat-sessions in guided internet-based cognitive behavioral therapy for adolescents with anxiety: a factorial design study. Front. Psych..

[bb0065] Berger T., Caspar F., Richardson R., Kneubühler B., Sutter D., Andersson G. (2011). Internet-based treatment of social phobia: a randomized controlled trial comparing unguided with two types of guided self-help. Behav. Res. Ther..

[bb0070] Berger T., Hohl E., Caspar F. (2009). Internet-based treatment for social phobia: a randomized controlled trial. J. Clin. Psychol..

[bb0075] Boettcher J., Berger T., Renneberg B. (2012). Does a pre-treatment diagnostic interview affect the outcome of internet-based self-help for social anxiety disorder? A randomized controlled trial. Behav. Cogn. Psychother..

[bb0080] Boettcher J., Carlbring P., Renneberg B., Berger T. (2013). Internet-based interventions for social anxiety disorder—an overview. Verhaltenstherapie.

[bb0085] Bur O.T., Krieger T., Moritz S., Klein J.P., Berger T. (2021). Optimizing the context of support to improve outcomes of internet-based self-help in individuals with depressive symptoms: protocol for a randomized factorial trial. JMIR Res. Protoc..

[bb0090] Carlbring P., Andersson G., Cuijpers P., Riper H., Hedman-Lagerlöf E. (2018). Internet-based vs. Face-to-Face cognitive behavior therapy for psychiatric and somatic disorders: an updated systematic review and meta-analysis. Cogn. Behav. Ther..

[bb0095] Chen H., Rodriguez M.A., Qian M., Kishimoto T., Lin M., Berger T. (2020). Predictors of treatment outcomes and adherence in internet-based cognitive behavioral therapy for social anxiety in China. Behav. Cogn. Psychother..

[bb0100] Clark D.M., Crozier W.R., Alden L.E. (2001). International Handbook of Social Anxiety: Concepts, Research and Intervention Relating to the Self and Shyness.

[bb0105] Clark D.M., Wells A., Heimberg R.G., Liebowitz M.R., Hope D., Schneider F. (1995). Social Phobia: Diagnosis, Assessment, and Treatment.

[bb0110] Clark D.M., Wells A., Salkoviskis P., Hackmann A. (1995).

[bb0115] Collins L.M. (2018).

[bb0120] Collins L.M., Dziak J.J., Kugler K.C., Trail J.B. (2014). Factorial experiments: efficient tools for evaluation of intervention components. Am. J. Prev. Med..

[bb0125] Dalrymple K.L., Zimmerman M. (2011). Treatment-seeking for social anxiety disorder in a general outpatient psychiatry setting. Psychiatry Res..

[bb0135] Donkin L., Christensen H., Naismith S.L., Neal B., Hickie I.B., Glozier N. (2011). A systematic review of the impact of adherence on the effectiveness of e-therapies. J. Med. Internet Res..

[bb0140] Duval S., Wicklund R.A. (1972). A Theory of Objective Self Awareness.

[bb0145] Eysenbach G., Consort-Ehealth Group (2011). CONSORT-EHEALTH: improving and standardizing evaluation reports of web-based and mobile health interventions. J. Med. Internet Res..

[bb0150] Faul F., Erdfelder E., Lang A.-G., Buchner A. (2007). G*power 3: a flexible statistical power analysis program for the social, behavioral, and biomedical sciences. Behav. Res. Methods.

[bb0155] Fehm L., Pelissolo A., Furmark T., Wittchen H.-U. (2005). Size and burden of social phobia in Europe. Eur. Neuropsychopharmacol..

[bb0160] Fenigstein A., Scheier M.F., Buss A.H. (1975). Public and private self-consciousness: assessment and theory. J. Consult. Clin. Psychol..

[bb0165] Feske U., Chambless D.L. (1995). Cognitive behavioral versus exposure only treatment for social phobia: a meta-analysis. Behav. Ther..

[bb0170] Filipp S.H., Freudenberg E. (1989).

[bb0175] Furukawa T.A., Suganuma A., Ostinelli E.G., Andersson G., Beevers C.G., Shumake J., Berger T., Boele F.W., Buntrock C., Carlbring P., Choi I., Christensen H., Mackinnon A., Dahne J., Huibers M.J.H., Ebert D.D., Farrer L., Forand N.R., Strunk D.R., Cuijpers P. (2021). Dismantling, optimising, and personalising internet cognitive behavioural therapy for depression: a systematic review and component network meta-analysis using individual participant data. Lancet Psychiatry.

[bb0180] Gandek B., Ware J.E., Aaronson N.K., Apolone G., Bjorner J.B., Brazier J.E., Bullinger M., Kaasa S., Leplege A., Prieto L., Sullivan M. (1998). Cross-validation of item selection and scoring for the SF-12 health survey in nine countries: results from the IQOLA project. International quality of life assessment. J. Clin. Epidemiol..

[bb0185] Gil P.J.M., Carrillo F.X.M., Meca J.S. (2001). Effectiveness of cognitive-behavioral treatment in social phobia: a meta-analytic review. Psychol. Spain.

[bb0190] Gould R.A., Buckminster S., Pollack M.H., Otto M.W., Massachusetts L.Y. (1997). Cognitive-behavioral and pharmacological treatment for social phobia: a meta-analysis. Clin. Psychol. Sci. Pract..

[bb0195] Gräfe K., Zipfel S., Herzog W., Löwe B. (2004). Screening psychischer Störungen mit dem “Gesundheitsfragebogen für patienten (PHQ-D)”: ergebnisse der deutschen validierungsstudie. [Screening for psychiatric disorders with the patient health questionnaire (PHQ). Results from the german validation study.]. Diagnostica.

[bb0205] Harris P.A., Taylor R., Minor B.L., Elliott V., Fernandez M., O’Neal L., McLeod L., Delacqua G., Delacqua F., Kirby J., Duda S.N. (2019). The REDCap consortium: building an international community of software platform partners. J. Biomed. Inform..

[bb0210] Harris P.A., Taylor R., Thielke R., Payne J., Gonzalez N., Conde J.G. (2009). Research electronic data capture (REDCap)—a metadata-driven methodology and workflow process for providing translational research informatics support. J. Biomed. Inform..

[bb0215] Hedman E., Andersson E., Ljótsson B., Andersson G., Rück C., Lindefors N. (2011). Cost-effectiveness of internet-based cognitive behavior therapy vs. cognitive behavioral group therapy for social anxiety disorder: results from a randomized controlled trial. Behav. Res. Ther..

[bb0220] Hedman E., Botella C., Berger T., Lindefors N., Andersson G. (2016). Guided Internet-Based Treatments in Psychiatry.

[bb5000] Hinz A., Blaser G., Schmutzer G., Bailer H., Grulke N., Brähler E., Albani C. (2010). Überprüfung und Normierung des “Fragebogen zur Erfassung dispositionaler Selbstaufmerksamkeit”(SAM) an einer repräsentativen deutschen Bevölkerungsstichprobe. Klinische Diagnostik Und Evaluation.

[bb0225] Holmes E.A., Craske M.G., Graybiel A.M. (2014). Psychological treatments: a call for mental-health science. Nat. News.

[bb0230] Hope D.A., Heimberg R.G., Bruch M.A. (1995). Dismantling cognitive-behavioral group therapy for social phobia. Behav. Res. Ther..

[bb0235] Hoyer J., Margraf J., Hoyer J., Margraf J. (2003). Angstdiagnostik: Grundlagen und Testverfahren.

[bb0240] Jacobson N.S., Truax P. (1991). Clinical significance: a statistical approach to defining meaningful change in psychotherapy research. J. Consult. Clin. Psychol..

[bb0245] Kählke F., Berger T., Schulz A., Baumeister H., Berking M., Auerbach R.P., Bruffaerts R., Cuijpers P., Kessler R.C., Ebert D.D. (2019). Efficacy of an unguided internet-based self-help intervention for social anxiety disorder in university students: a randomized controlled trial. Int. J. Methods Psychiatr. Res..

[bb0250] Karyotaki E., Riper H., Twisk J., Hoogendoorn A., Kleiboer A., Mira A., Mackinnon A., Meyer B., Botella C., Littlewood E., Andersson G., Christensen H., Klein J.P., Schröder J., Bretón-López J., Scheider J., Griffiths K., Farrer L., Huibers M.J.H., Cuijpers P. (2017). Efficacy of self-guided internet-based cognitive behavioral therapy in the treatment of depressive symptoms: a meta-analysis of individual participant data. JAMA Psychiatry.

[bb0255] Kazdin A.E. (2007). Mediators and mechanisms of change in psychotherapy research. Annu. Rev. Clin. Psychol..

[bb0260] Kazdin A.E. (2017). Addressing the treatment gap: a key challenge for extending evidence-based psychosocial interventions. Behav. Res. Ther..

[bb0265] Kishimoto T., Krieger T., Berger T., Qian M., Chen H., Yang Y. (2016). Internet-based cognitive behavioral therapy for social anxiety with and without guidance compared to a wait list in China: a propensity score study. Psychother. Psychosom..

[bb0270] Klein J.P., Berger T., Schröder J., Späth C., Meyer B., Caspar F., Lutz W., Arndt A., Greiner W., Gräfe V., Hautzinger M., Fuhr K., Rose M., Nolte S., Löwe B., Andersson G., Vettorazzi E., Moritz S., Hohagen F. (2016). Effects of a psychological internet intervention in the treatment of mild to moderate depressive symptoms: results of the EVIDENT study, a randomized controlled trial. Psychother. Psychosom..

[bb0275] Kraemer H.C., Wilson G.T., Fairburn C.G., Agras W.S. (2002). Mediators and moderators of treatment effects in randomized clinical trials. Arch. Gen. Psychiatry.

[bb0280] Kroenke K., Spitzer R.L., Williams J.B.W., Löwe B. (2010). The patient health questionnaire somatic, anxiety, and depressive symptom scales: a systematic review. Gen. Hosp. Psychiatry.

[bb0285] Ladwig I., Rief W., Nestoriuc Y. (2014). What are the risks and side effects of psychotherapy? - Development of an inventory for the assessment of negative effects of psychotherapy (INEP). Verhaltenstherapie.

[bb0290] Lin L.-Y., Wang K., Kishimoto T., Rodriguez M., Qian M., Yang Y., Zhao Q., Berger T., Tian C. (2020). An internet-based intervention for individuals with social anxiety and different levels of taijin kyofusho in China. J. Cross-Cult. Psychol..

[bb0295] Loerinc A.G., Meuret A.E., Twohig M.P., Rosenfield D., Bluett E.J., Craske M.G. (2015). Response rates for CBT for anxiety disorders: need for standardized criteria. Clin. Psychol. Rev..

[bb0300] Löwe B., Decker O., Müller S., Brähler E., Schellberg D., Herzog W., Herzberg P.Y. (2008). Validation and standardization of the generalized anxiety disorder screener (GAD-7) in the general population. Med. Care.

[bb0305] Löwe B., Kroenke K., Herzog W., Gräfe K. (2004). Measuring depression outcome with a brief self-report instrument: sensitivity to change of the patient health questionnaire (PHQ-9). J. Affect. Disord..

[bb0310] Mattick R.P., Clarke J.C. (1998). Development and validation of measures of social phobia scrutiny fear and social interaction anxiety. Behav. Res. Ther..

[bb0315] Mattick R.P., Peters L., Clarke J.C. (1989). Exposure and cognitive restructuring for social phobia: a controlled study. Behav. Ther..

[bb0325] Nortje C., Posthumus T. (2012). Scores on an emotional stroop task after treatment of social anxiety disorder. Psychol. Rep..

[bb0330] Powers M.B., Sigmarsson S.R., Emmelkamp P.M.G. (2008). A meta-analytic review of psychological treatments for social anxiety disorder. Int. J. Cogn. Ther..

[bb0335] Schmidt J., Wittmann W.W., Brähler E., Schumacher J., Strauß B. (2002). Diagnostische verfahren in der psychotherapie.

[bb0340] Schulz A., Stolz T., Vincent A., Krieger T., Andersson G., Berger T. (2016). A sorrow shared is a sorrow halved? A three-arm randomized controlled trial comparing internet-based clinician-guided individual versus group treatment for social anxiety disorder. Behav. Res. Ther..

[bb0345] Sheehan D.V., Lecrubier Y., Harnett Sheehan K., Janavs J., Weiller E., Keskiner A., Schinka J., Knapp E., Sheehan M.F., Dunbar G. (1997). The validity of the Mini international neuropsychiatric interview (MINI) according to the SCID-P and its reliability. Eur. Psychiatry.

[bb0350] Sheehan D.V., Lecrubier Y., Sheehan K.H., Amorim P., Janavs J., Weiller E., Hergueta T., Baker R., Dunbar G.C. (1998). The Mini-International Neuropsychiatric Interview (M.I.N.I.): the development and validation of a structured diagnostic psychiatric interview for DSM-IV and ICD-10. J. Clin. Psychiatry.

[bb0355] Snow G. (2020). blockrand: randomization for block random clinical trials (1.5) [computer software]. https://CRAN.R-project.org/package=blockrand.

[bb0360] Song M.-K., Lin F.-C., Ward S.E., Fine J.P. (2013). Composite variables: when and how. Nurs. Res..

[bb0365] Spitzer R.L., Kroenke K., Williams J.B. (1999). Validation and utility of a self-report version of PRIME-MD: the PHQ primary care study. Primary care evaluation of mental disorders. Patient health questionnaire. JAMA.

[bb0370] Spitzer R.L., Kroenke K., Williams J.B.W., Löwe B. (2006). A brief measure for assessing generalized anxiety disorder: the GAD-7. Arch. Intern. Med..

[bb0375] Stangier U., Heidenreich T. (2004). Internationale Skalen für Psychiatrie.

[bb0380] Stangier U., Heidenreich T., Berardi A., Golbs U., Hoyer J. (1999). Die erfassung sozialer phobie durch die social interaction anxiety scale (SIAS) und die social phobia scale (SPS). Z. Klin. Psychol. Psychother..

[bb0385] Stangier U., Heidenreich T., Ehlers A., Clark D.M. (1996).

[bb0390] Stangier U., Heidenreich T., Ehlers A., Clark D.M. (1996).

[bb0395] Stein D.J., Lim C.C.W., Roest A.M., de Jonge P., Aguilar-Gaxiola S., Al-Hamzawi A., Alonso J., Benjet C., Bromet E.J., Bruffaerts R., de Girolamo G., Florescu S., Gureje O., Haro J.M., Harris M.G., He Y., Hinkov H., Horiguchi I., Hu C., WHO World Mental Health Survey Collaborators (2017). The cross-national epidemiology of social anxiety disorder: data from the World Mental Health Survey Initiative. BMC Med..

[bb0400] Stolz T., Schulz A., Krieger T., Vincent A., Urech A., Moser C., Westermann S., Berger T. (2018). A mobile app for social anxiety disorder: a three-arm randomized controlled trial comparing mobile and PC-based guided self-help interventions. J. Consult. Clin. Psychol..

[bb0405] Ware J.E., Kosinski M., Keller S.D. (1996). A 12-item short-form health survey: construction of scales and preliminary tests of reliability and validity. Med. Care.

[bb0410] Watkins E.R., Newbold A. (2020). Factorial designs help to understand how psychological therapy works. Front. Psych..

[bb0415] Watkins E.R., Newbold A., Tester-Jones M., Javaid M., Cadman J., Collins L.M., Graham J., Mostazir M. (2016). Implementing multifactorial psychotherapy research in online virtual environments (IMPROVE-2): study protocol for a phase III trial of the MOST randomized component selection method for internet cognitive-behavioural therapy for depression. BMC Psychiatry.

[bb0420] Wells A., Stopa L., Clark D.M. (1993).

